# Oral health profile status and treatment needs in the Salvadoran elderly population: a cross-sectional study

**DOI:** 10.1186/s12903-022-02278-z

**Published:** 2022-06-21

**Authors:** Guillermo Alfonso Aguirre Escobar, Ruth Fernández de Quezada, Wendy Yesenia Escobar de González, Katleen Argentina Aguirre de Rodríguez, Ángel Gil de Miguel, Francisco José Rivas Cartagena

**Affiliations:** 1 Epidemiology and Public Health Program, University Rey Juan Carlos, 28933 Madrid, Spain; 2 Research Center, Faculty of Dentistry, University of El Salvador, 01101 San Salvador, El Salvador; 3 Department of Preventive Medicine and Public Health, University Rey Juan Carlos, 28933 Madrid, Spain

**Keywords:** Elderly population, Epidemiological profile, Treatment need

## Abstract

**Introduction:**

Older adults are a highly vulnerable group in their general health condition, including oral health that can be influenced by different factors, among them, changes in oral tissues inherent to the physiological processes of aging and by systemic condition. In El Salvador, it is a group that has received little attention at the public health level.

**Objective:**

To determine the profile of the oral health status and treatment needs of the elderly population in El Salvador.

**Materials and methods:**

Secondary cross-sectional analysis of data from the last oral health survey in 471 Salvadorans aged 60 years and older. The variables under study were: sociodemographics, brushing frequency, oral hygiene according to simplified oral hygiene index (OHI-S), caries experience according to decayed, missing, and filled teeth index (DMFT) modified with international caries detection and assessment system (ICDAS) criteria, periodontal status through the community periodontal index of treatment needs (CPITN), edentulism and treatment needs. Statistical analysis was conducted using chi-square test, ANOVA, z-test and linear regression (*p* < 0.05).

**Results:**

The older adults presented poor oral hygiene, low brushing frequency, high tooth loss with an average of 16 missing teeth while one third presented total edentulism. Most of the older adults were categorized as having "poor or very Poor" oral hygiene. Almost all respondents presented some degree of periodontal disease and required restorative intervention.

**Conclusion:**

The oral health status of elderly Salvadoran is poor. Furthermore, the development of public policies and specific oral health strategies aimed at this population is urgent.

## Introduction

In the world, the absolute number and proportion of older adults in society have increased in recent decades and will continue to do so in the coming years. This phenomenon is also occurring in El Salvador, where, according to the latest census of 2007, the older adult population is 745,874, representing 11.22% of the total population [1]. According to the World Health Organization (WHO), the aging index in the country during the period from 2012 to 2015 grew from 34.3 to 38.8%; in fact, in the same period, the life expectancy at birth increased from 72.1 to 72.7 years [2].

The elderly population is considered highly vulnerable and is affected by multiple systemic diseases, including oral diseases, which have the potential to affect the quality of life due to the problems they generate in chewing, swallowing and communication [3, 4]. Caries and periodontal diseases are the oral conditions most frequently associated with tooth loss in this population [5, 6]. Furthermore, society in general has the misperception that oral deterioration is normal and inevitable in old age. In addition, studies report that poor oral health increases the risk of systemic disease and severity states by COVID-19 [7, 8].

Despite being a vulnerable group, there are few studies in El Salvador that address the situation of older adults; therefore, oral health policies in the country do not prioritize this population, being marginalized from the programs; even the dental curricula in the country include minimal content on the diagnosis and care of older adults.

The Ministry of Health of El Salvador, in the Technical Standard for Comprehensive Health Care, provides guidelines for oral health care of the elderly; however, the diagnosis only includes aspects oriented to edentulous patients, including whether they are totally or partially edentulous, whether they use any type of dental prosthesis, and whether there is any soft tissue affection, pain and halitosis, but there is no specific program for comprehensive oral care [9]. However, the older adult presents changes in the lining tissue, salivary function, dental tissue, periodontal tissue, joint disorders, occlusal changes, changes in bone tissue, taste alterations, among others [10, 11].

Therefore, the purpose of this study was to determine the profile of oral health status and treatment needs of the Salvadoran elderly population in order to provide data that could serve as a basis for the creation of future oral health care programs that respond to the needs detected in this susceptible population group. Also, for dental schools to emphasize the components of oral health care for the elderly.

## Materials and methods

### Study design and setting

This study complies with the Strengthening the Reporting of Observational Studies in Epidemiology (STROBE) guidelines [12]. The study comprised a secondary analysis of data from the latest population survey on oral health in El Salvador, which was carried out using pathfinder methodology according to the WHO, with age ranges divided into 60–75 years and over 75 years considering the life expectancies of Salvadorans, to establish differences between the two groups. The total population examined in the survey totaled 3881, with 471 subjects aged 60 years or older from 24 municipalities. The population considered in this study was recruited in public health centers, nursing homes, churches, among others, and their participation was voluntary [13].

The sample was obtained using the stratified cluster sampling technique of the WHO exploratory method, with a modification that consisted of extending the age to guarantee the inclusion of vulnerable groups in order to demonstrate their declining oral health status, which had been observed in the clinical care provided in public health services, and that these would serve to recommend changes in their policies and care programs to the competent authorities [14].

### Measures

Prior to data collection, 10 theoretical and practical workshops and 1 pilot study were carried out in order to unify criteria, test instruments, establish average times, achieve adequate consistency in the diagnoses and data records. As established in the protocol for the unification of criteria for the diagnosis of dental caries according to the International Caries Detection and Assessment System (ICDAS), the statistical analysis of the concordance of the surfaces evaluated in the Epidat 3.1 program was carried out through Cohen's Kappa Coefficient, obtaining a concordance of 0.84 kappa value.

Specific instruments for each variable and measurement indexes were used to record the data. The independent variables were: sex, age, residence, region and schooling. The dependent variables were: Brushing frequency, oral hygiene according to Simplified Oral Hygiene Index (OHI-S) [15], caries experience according to Decayed, Missing, and Filled Teeth index (DMFT) modified with ICDAS criteria, periodontal status through the Community Periodontal Index of Treatment Needs (CPITN) and the main treatment needs of this population.

### Clinical examination

All respondents signed an informed consent declaration for the clinical examination performed by one of the 6 dentists qualified and calibrated for this survey. Mirror #5, WHO probe and healing forceps were used; for illumination, in addition to the environmental (natural and/or artificial), a miner type lamp with concentrated beam and average power of 0.072 watts was used; humidity was controlled with relative isolation with cotton rolls and gauze; then, the diagnosis was performed following the observation guide.

The examiner made the clinical diagnosis of caries by recording the caries experience according to the DMFT index modified with ICDAS criteria. The presence of dental plaque (with OHI-S criteria) and periodontal tissues (using CPITN) were examined, recording the findings in the respective sections of the guide. Edentulism status was determined and the main treatment needs were established.

### Statistical analysis

The statistical analyses were performed using SPSS version 25.0 (SPSS Inc., Chicago, IL, USA). Frequencies, means, significance and statistical inference were obtained using descriptive statistics, z-test or test of proportions, analysis of variance (ANOVA) and χ^2^. Multivariate analysis was performed with linear regression and statistical significance was set at *p* < 0.05.

## Results

Of the 471 older adults in this study, 47.5% were male and 50.5% were female. In total, 60.1% of the older adults lived in an urban area and central El Salvador was the most-represented region (21.4%). Most of the older adults (63.4%) had some kind of schooling, though 36.5% did not (Table 1).

**Table 1:** Sociodemographic characteristics of the study participants

Variable	*n* = 471	
	*n* %	
*Sex*		
Male	233	49.5
Female	238	50.5
*Age*		
From 60 to 75 years old	320	67.94
Over 75 years old	151	32.06
*Residence*		
Urban	283	60.1
Rural	188	39.9
*Region*		
Central	101	21.4
Occidental	79	16.7
Oriental	59	12.5
North	90	19.1
Paracentral	56	11.8
Coast	86	18.2
*Schooling*		
No schooling	172	36.5
First cycle	133	28.2
Second cycle	81	17.2
Third cycle	41	8.7
Baccalaureate	28	5.9
Top	16	3.4

Source: Own elaboration

According to the chi-squared statistical test and the z-test or proportions test, there were significant differences between men and women, with women brushing more frequently. For the age variable, statistically significant differences were found, with people under 76 years of age brushing more frequently. There was also a significant difference, according to region, with the majority of people in the east brushing more frequently (Table 2).

**Table 2:** Frequency of brushing in the elderly

Variables	Category	n (%)	After every meal (i.e., three times a day)	Once or twice a day (i.e., morning and/or evening)	Never/almost never/occasionally/don’t know	*p* value
			*n* (%)	*n* (%)	*n* (%)	
60 years and older		*n* = 471 (100)	135 (28.66)	245 (52.02)	91 (19.32)	
Sex	Male	233 (100)	50 (21.46)	129 (55.36)	54 (23.18)⋆⋆	0.003*
	Female	238 (100)	85 (35.71)	116 (48.74)	37 (15.55)	
Age	From 60 to 75 years old	320 (100)	103 (32.19)	172 (53.75)	45 (14.06)	0.000*
	Over 75 years old	151 (100)	32 (21.19)	73 (48.34)	46 (30.46)⋆⋆	
Residence	Urban	283 (100)	77 (27.21)	149 (52.65)	57 (20.14)	0.662
	Rural	188 (100)	58 (30.85)	96 (51.06)	34 (18.09)	
Region	Central	101 (100)	16 (15.84)	59 (58.42)⋆⋆	26 (25.74)	0.005*
	Occidental	79 (100)	23 (29.11)	43 (54.43)	13 (16.46)	
	Oriental	59 (100)	29 (49.15)	24 (40.68)	6 (10.17)	
	North	90 (100)	28 (31.11)	41 (45.56)	21 (23.33)	
	Paracentral	56 (100)	11 (19.64)	32 (57.14)	13 (23.21)	
	Coast	86 (100)	28 (32.56)	46 (53.49)	12 (13.95)	

Source: Own elaboration

^*^Significant. ⋆⋆ Z-test

We found that 42.68% of Salvadoran older adults presented poor oral hygiene (very bad category), which mainly affected men as well as older adults residing in urban areas, specifically in the central region of El Salvador. Statistically significant differences were found for the variables sex and region (Table 3).

**Table 3:** Oral hygiene level by sex, residence and region

Oral hygiene level	*n*	Optimum (%)	Regular (%)	Bad (%)	Very bad (%)	*p* value
*Sex*						
Male	233 (49.47)	44 (9.34)	31 (6.58)	38 (8.07)	120 (25.48)	0.001*
Female	238 (50.53)	74 (15.71)	40 (8.49)	43 (9.13)	81 (17.2)	
*Age*						
From 60 to 75 years old	320 (67.94)	77 (16.35)	56 (11.89)	56 (11.89)	131 (27.81)	0.168
Over 75 years old	151 (32.06)	41 (8.7)	15 (3.18)	25 (5.31)	70 (14.86)	
*Residence*						
Urban	283 (60.08)	72 (15.29)	42 (8.92)	47 (9.98)	122 (25.9)	0.97
Rural	188 (39.92)	46 (9.77)	29 (6.16)	34 (7.22)	79 (16.77)	
*Region*						
Central	101 (21.44)	4 (0.85)	9 (1.91)	18 (3.82)	70 (14.86)	0.000*
Occidental	79 (16.77)	12 (2.55)	16 (3.4)	18 (3.82)	33 (7.01)	
Oriental	59 (12.53)	32 (6.79)	7 (1.49)	12 (2.55)	8 (1.7)	
North	90 (19.11)	35 (7.43)	17 (3.61)	7 (1.49)	31 (6.58)	
Paracentral	56 (11.89)	14 (2.97)	10 (2.12)	12 (2.55)	20 (4.25)	
Coast	86 (18.26)	21 (4.46)	12 (2.55)	14 (2.97)	39 (8.28)	
TOTAL	471	118 (25.5)	71 (15.07)	81 (17.2)	201 (42.68)	

Source: Own elaboration

^*^Significant

With regard to caries experience, a high DMFT index of 23.38 was determined, with the “Missing” component being the one that raised the index figure in both men and women. In the bivariate analysis, significant differences were found in caries experience (DMFT) with respect to age and region. For the multivariate analysis, significant differences were found in tooth loss with respect to age (Table 4).

**Table 4:** DMFT index modified with ICDAS criteria associated with sociodemographic factors

	Bivariate analysis									Multivariate analysis for missing teeth		
	*n*	D	*p* value	M	*p* value	F	*p* value	DMFT (Standar Deviation)	*p* value	Estimation	*p* value	(95% CI)
60 years of age or older	471	5.92		16.18		1.28		23.38 (0.26)				
*Sex*												
Male	233	6.85	0.001*	15.32	0.063	1.25	0.808	23.36 (0.38)	0.1604	2.312	0.01	(0.555, 4.069)
Female	238	5		17.02		1.32		23.24 (0.37)				
Age*												
From 60 to 75 years old	320	6.18	0.180	14.44	0.000*	1.69	0.000*	22.23 (0.33)	0.000*	5.835	0.000*	(3.955, 7.716)
Over 75 years old	151	5.36		19.86		0.43		25.56 (0.37)				
*Residence*												
Urban	283	6.42	0.031	16.19	0.973	1.22	0.579	23.78 (0.31)	0.1419	0.262	0.792	(− 1.689, 2.213)
Rural	188	5.16		16.16		1.38		22.57 (0.47)				
Region*												
Central	101	7.9	0.000*	13.81	0.000*	1.48	0.242	23.18 (0.51)	0.0065*	0.144	0.59	(− 0.382, 0.671)
Occidental	79	6.2		16.06		1.08		23.16 (0.64)				
Oriental	59	5.75		18.51		0.56		24.81 (0.63)				
North	90	2.74		20.30		1.12		24.07 (0.65)				
Paracentral	56	7.25		14.82		1.45		23.27 (0.70)				
Coast	86	5.9		14.03		1.81		21.74 (0.71)				

Source: Own elaboration

^*^Significant

Regarding the main treatment needs, almost all of the Salvadoran population aged 60 years and older required at least one prosthetic treatment (90.23%), followed by fillings (46.28%) and exodontia (40.55%). The need for exodontia treatment is related to the sociodemographic variables sex (*p* = 0.001), age (*p* = 0.000), residence (*p* = 0.002) and region (*p* = 0.000). In the multivariate analysis, only the age variable was found to be influential in the model, given that as the age of the subject increases in years, the probability of needing dental prostheses increases by 5.835 (Table 5).

**Table 5:** Treatment needs associated with sociodemographic factors

Variable	Bivariate analysis													Multivariate analysis for dental prosthesis		
	n	Fillings			Prosthesis			Pulp treatment			Exodontia			Estimation	*p* value	(95% C.I)
		%	Media	*p* value	%	Media	*p* value	%	Media	*p* value	%	Media	*p* value			
*Sex*																
Male	233	24.42	3.23	0.97	45.86	13.52	0.731	10.4	0.65	0.001*	23.89	2.85	0.001*	2.312	0.01	(0.555, 4.069)
Female	238	21.87	2.34		44.37	13.84		7.43	0.22		16.56	1.65				
*Age*																
From 60 to 75 years old	320	36.09	3.23	0.014	60.72	12.14	0.000*	14.23	0.5	0.142	23.57	1.78	0.000*	5.835	0.000*	(3.955, 7.716)
Over 75 years old	151	10.19	1.81		29.51	16.93		3.61	0.29		16.99	3.24				
*Residence*																
Urban	283	27.81	2.92	0.526	55.63	14.27	0.526	11.25	0.43	0.978	30.36	2.73	0.002*	0.262	0.792	(− 1.689, 2.213)
Rural	188	18.47	2.57		34.61	12.8		6.58	0.43		10.19	1.51				
*Region*																
Central	101	11.46	2.62*	0.120	19.75	12.26	0.000*	4.67	0.6	0.406	13.59	4.06	0.000*	0.144	0.59	(− 0.382, 0.671)
Occidental	79	8.7	2.87		14.44	11.49		2.97	0.28		6.37	1.96				
Oriental	59	6.79	3.85		12.53	18.37		2.55	0.51		4.25	1.64				
North	90	4.88	1.33		16.56	16.98		1.91	0.21		4.25	1				
Paracentral	56	5.1	3.39		11.46	14.2		3.4	0.46		7.01	2.87				
Coast	86	9.34	3.26		15.5	10.35		2.34	0.53		5.1	1.67				
Total	471	46.28	2.78		90.23	13.68		17.83	0.43		40.55	2.24				

Source: Own elaboration

^*^Significant

Periodontopathies were evaluated through the CPITN, determining a prevalence (65.4%) of periodontal disease in older adults, who required dental prophylaxis, oral hygiene intervention, professional scaling and periodontal surgery. Finally, it was determined that 31.42% of the older adults were edentulous, predominantly women and respondents from eastern El Salvador (Table 6).

**Table 6:** Community periodontal index of treatment needs (CPITN) according to the maximum value that each patient presented

CPITN	*n*	0	1	2	3	4	Edentulous	*p* value
60 and over	471	3.18	6.37	23.57	23.57	11.89	31.42	
*Sex*								
Male	233	5 (1.06)	9 (1.91)	57 (12.1)	68 (14.44)	30 (6.37)	64 (13.59)	0.010
Female	238	10 (2.12)	21 (4.46)	54 (11.46)	43 (9.13)	26 (5.52)	84 (17.83)	
*Age*								
From 60 to 75 years old	320	10 (2.12	25 (5.31)	85 (18.05)	78 (16.56)	41 (8.7)	81 (17.2)	0.001*
Over 75 years old	151	5 (1.06)	5 (1.06)	26 (5.52)	33 (7.01)	15 (3.18)	67 (14.23)	
*Residence*								
Urban	283	10 (2.12)	16 (3.4)	76 (16.14)	64 (13.59)	36 (7.64)	81 (17.2)	0.256
Rural	188	5 (1.06)	14 (2.97)	35 (7.43)	47 (9.98)	20 (4.25)	67 (14.23)	
*Region*								
Central	101	0	4 (0.85)	24 (5.21)	42 (8.92)	14 (2.97)	17 (3.61)	0.000*
Occidental	79	0	8 (1.7)	22 (4.67)	23 (4.88)	11 (2.34)	15 (3.18)	
Oriental	59	3 (0.64)	1 (0.21)	25 (5.31)	6 (1.27)	0	24 (5.1)	
North	90	6 (1.27)	8 (1.7)	10 (2.12)	5 (1.06)	8 (1.7)	53 (11.25)	
Paracentral	56	4 (0.85)	3 (0.64)	10 (2.12)	14 (2.97)	9 (1.91)	16 (3.4)	
Coast	86	2 (0.42)	6 (1.27)	20 (4.25)	21 (4.46)	14 (2.97)	23 (4.88)	

Source: Own elaboration

^*^Significant

## Discussion

The objective of the study was to determine the profile of the oral health status and treatment needs of the elderly population of El Salvador, an age group that is constantly increasing in the country and worldwide.

Of the total number of study subjects, one third have no schooling, similar to the study in China, where one third of the older adults have a low level of education, most of them being illiterate [16].

A low frequency of brushing was identified in this study, and significant differences were found between the variables sex and region. Similar to the results from El Salvador, Lu Liu et al. reported in northeastern China that one third of the elderly brush their teeth less than once a day [17]. In Poland, Wojciech Skorupka et al. report that the majority of older adults brush their teeth only 1 or 2 times a day (80%) [18]; these similarities lead to the inference that regardless of the cultural or developmental differences between countries, there are common factors that could influence the oral hygiene of this susceptible group, among these, the economic difficulty for the population of this age group to buy toothbrushes and toothpaste, prioritizing other needs such as food and medicines, together with the ingrained habits of older adults and the decrease in social life, since many of them are without productive activity, which forces them to spend most of the day in their own homes or in nursing homes for the elderly.

Consistent with the infrequency of brushing found in this study, it was also identified that more than half of Salvadoran older adults have "Poor or Very Poor" hygiene, similar to the results of other studies such as those conducted in India and Turkey in which deficient levels of oral hygiene were also reported [3, 19]. This condition worsens their oral health status, as well as the aging process, degree of physical disability, oral health assessment, access to health services, among others [20]. The results of the brushing frequency and oral hygiene variables are evidence of the need for educational-preventive care with methods that allow motivation to improve oral hygiene practices in older adults.

The DMFT index modified with ICDAS criteria reflects an average of 5.9 decayed teeth per individual, finding significant differences between sexes. This finding differ from the results of other studies such as those conducted in New Zealand, Turkey, Spain, Colombia, Belgium, China and France where fewer caries experiences in their active state were reported [16, 19, 21–25]. The difference in the results of our study could be primarily attributed in the first place to the criteria used to establish the diagnosis of caries, which, in our case, when using ICDAS, initial carious lesions such as the white spot were considered, while other studies that have used WHO criteria have only included cavitated carious lesions. Other factors that could be attributed to contributing to the difference could be the strength of the public health systems in developed countries, since in our country, health care programs prioritize children and pregnant women. It could also be inferred to the difference in diets, the economic, cultural and educational level of the populations surveyed in each country.

The mean number of teeth lost was 16.18, this component represents 69.20% of the total DMFT; according to the multivariate analysis, the trend is the greater the age, the greater the tooth loss. Our results are close to those reported in China, Spain, Belgium, Turkey and Colombia in which also the missing component represents a high percentage, between 71 and 86% [16, 19, 22–24] and different from those of Mexico and New Zealand that reported a lower percentage of missing teeth of approximately 50% of the DMFT index [21, 26]. Despite the differences in the results between countries, the data show that dental loss is a constant, representing a global public health problem that is yet to be solved due to its potential negative impact on the quality of life of those who suffer from it.

Almost one third of the older adults participating in the study are edentulous, affecting the ability to chew and subsequently the general state of health due to nutritional deficiencies as a result of the difficulty in eating; it also affects the function of speech, reduces self-esteem and impairs social integration. The same proportion of edentulous patients has been observed in other similar studies in Latin America such as those carried out in Brazil and Colombia and differing from the rate of total edentulism found in China, India, Ghana and South Africa [23, 27, 28]; the differences could be attributable to factors such as sociodemographic variables, genetic variants, dietary culture and the coverage provided by the dental care programs in each country.

In accordance with the high prevalence of missing teeth, almost all of the study subjects required prosthetic rehabilitation. Multivariate analysis showed that the higher the age, the higher the probability of needing prosthetic treatment, therefore, it is urgent at the public level to prioritize oral health care for older adults to improve their quality of life and masticatory function, thus reducing the risk of malnutrition in Salvadoran older adults [20, 29].

On the other hand, regarding periodontal health, the CPITN results indicated that the majority of the population needs periodontal treatments of professional scaling, prophylaxis and some type of periodontal surgery. These results also show the oral health deficiencies of this sector of the population and support the need to implement programs that encourage self-care and emphasize oral hygiene as soon as possible. Our results were similar to those obtained in other countries that used the same index, such as Turkey with 90% and Colombia with 93.4%, both of which reported that their population required some type of periodontal treatment [19, 23]. These data demonstrate that regardless of social, cultural or economic differences, periodontal disease is more severe in the elderly and therefore deserves to be prioritized among public health care needs.

A limitation of this study was its cross-sectional nature, since it does not allow establishing cause-effect relationships between the variables studied, so analytical studies are needed to establish the influence of the variables on oral health status. A recall bias also occurs, since it is known that in adulthood patients lose teeth also due to periodontal disease and it is impossible to determine the real reason for the indication of a periodontal.

## Conclusions

In general, the oral health status of elderly Salvadoran is poor. They report high levels of dental caries, periodontal disease and edentulism, accompanied by poor oral hygiene. Furthermore, the development of public policies and specific oral health strategies aimed at this vulnerable population is urgent.

## Data Availability

The datasets generated and analyzed during the current study are not publicly available due to ethical approval limitations involving anonymity but are available from the corresponding author on reasonable request.

## Abbreviations

ANOVA: Analysis of variance
CPITN: Community periodontal index of treatment needs
DMF-T index: Decayed, missing, filled-teeth index
ICDAS: International caries detection and assessment system
OHI-S: Simplified oral hygiene index
STROBE: Strengthening the reporting of observational studies in epidemiology
WHO: World Health Organization
